# Refractory multisystem sarcoidosis involving pelvic bone responding to infliximab

**DOI:** 10.1186/1471-2474-14-S1-A9

**Published:** 2013-02-14

**Authors:** Uma Karjigi, Anupam Paul

**Affiliations:** 1 James Cook University Hospitals NHS Trust, UK

## Background

Chronic progressive multisystem granulomatous disease is seen in 10–30% of patients with sarcoidosis and can result in end organ damage [1]. Corticosteroids are the mainstay of treatment with the addition of cytotoxic agents in severe cases. Some patients are refractory to such treatment and therefore management is a challenge. There is currently limited evidence for biological agents such as infliximab, a monoclonal anti-tumor necrosis factor-α antibody in the treatment of multisystem sarcoidosis. Aim of this case report is to disseminate knowledge concerning successful treatment of multisystem sarcoidosis involving pelvis with the use of TNF-α blockers in refractory cases.

## Case presentation

33 yr old lady referred to Rheumatology with arthralgia, myalgia, malaise, headaches, facial swelling, nausea, vomiting, ear and neck pain and tingling in her fingers. Her past medical history revealed uveitis. Subsequently she developed Right facial palsy and noted to have bilateral parotid enlargement. Chest X-ray and CT chest confirmed extensive mediastinal and bilateral hilar lymphadenopathy. Serum ACE levels were elevated and lymphnode biopsy confirmed Sarcoidosis.

**Figure 1:**
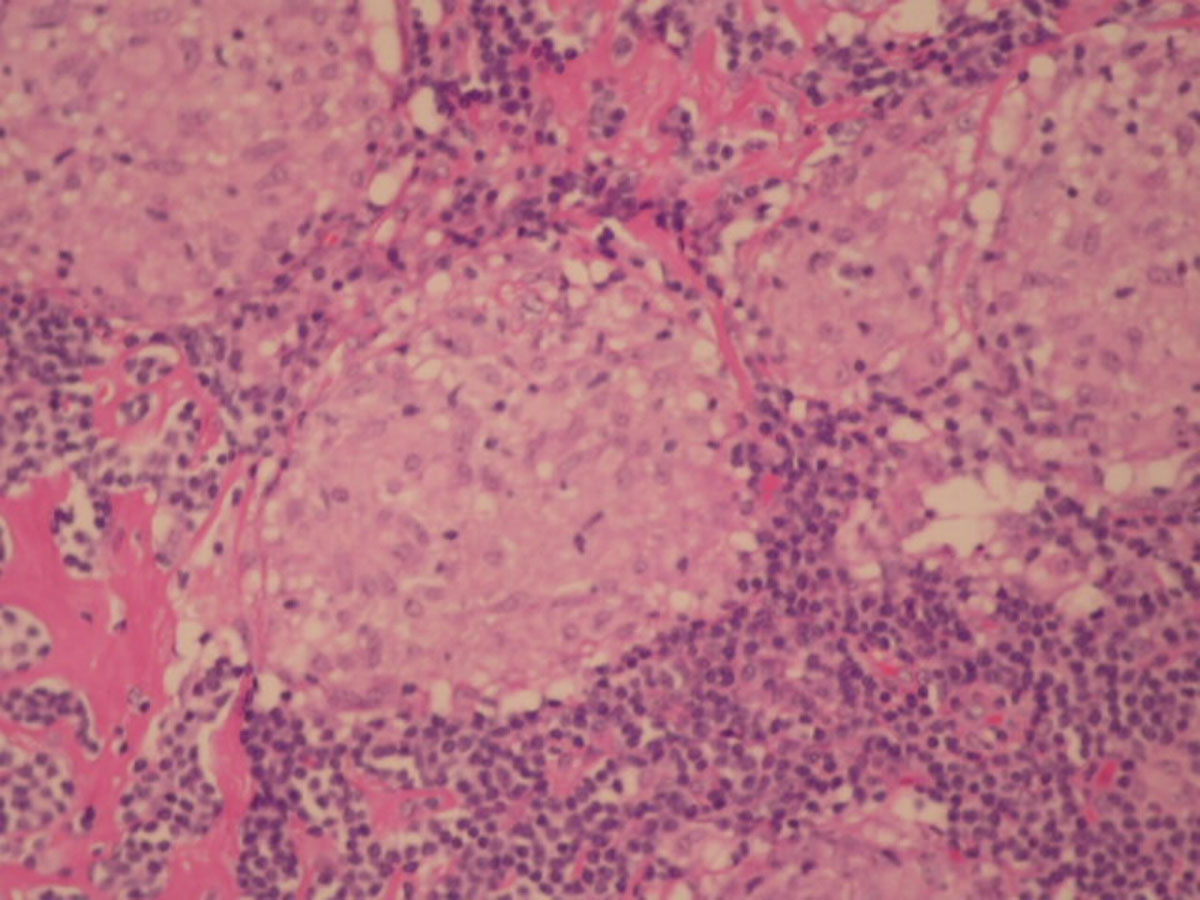
Cervical node biopsy showing Granulomas with little lymphoid tissue

She was started on oral prednisolone 40mg daily with good response. However she developed erythema nodosum, relapse of uveitis and repeated episodes of hypercalcemia needing hospital admission on trying to reduce the dose of steroids. She complained of pain in hip joints. MRI Pelvis showed multiple bony lesions and bone biopsy confirmed bony involvement of sarcoidosis.

**Figure 2:**
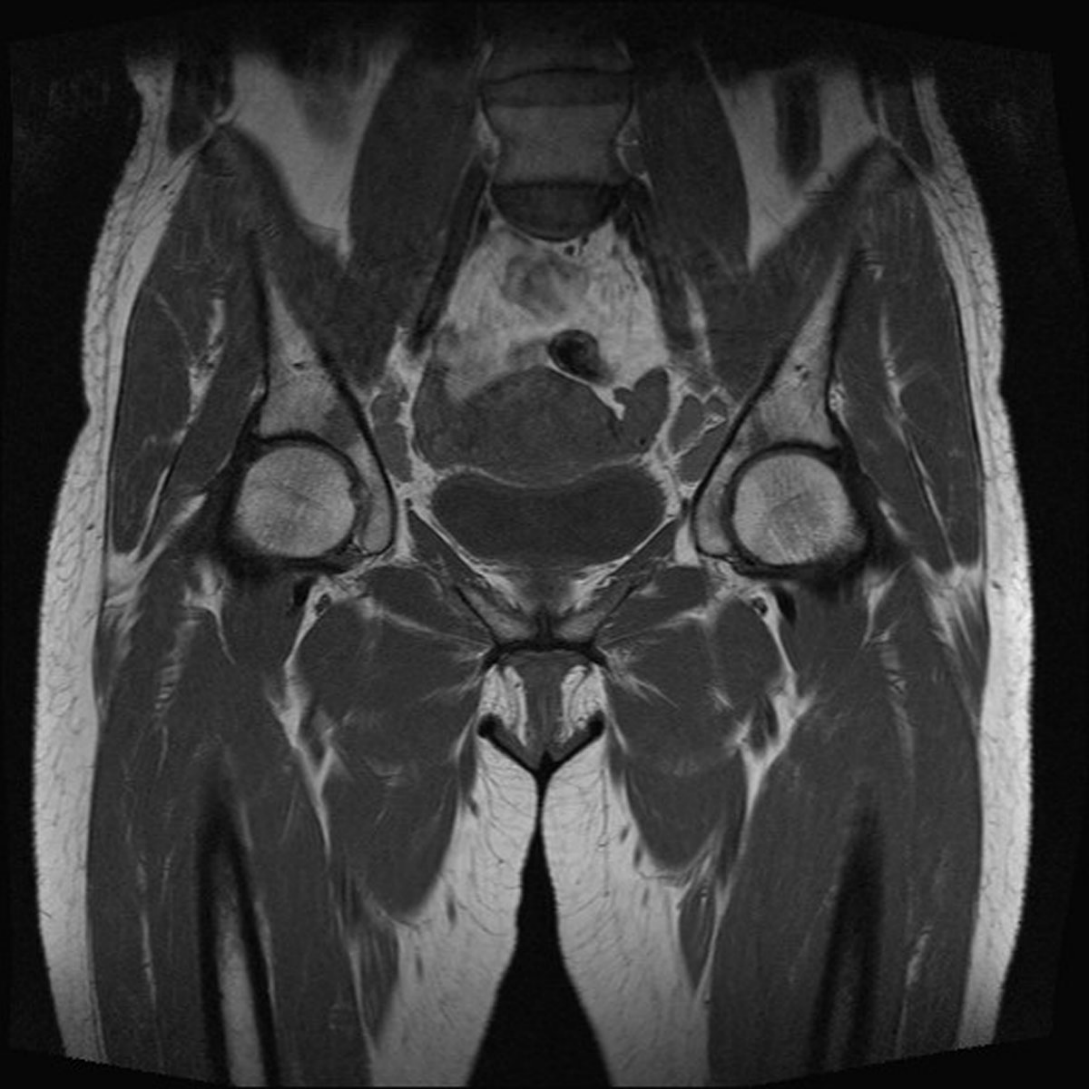
MRI pelvis showing lesions over pelvis

**Figure 3:**
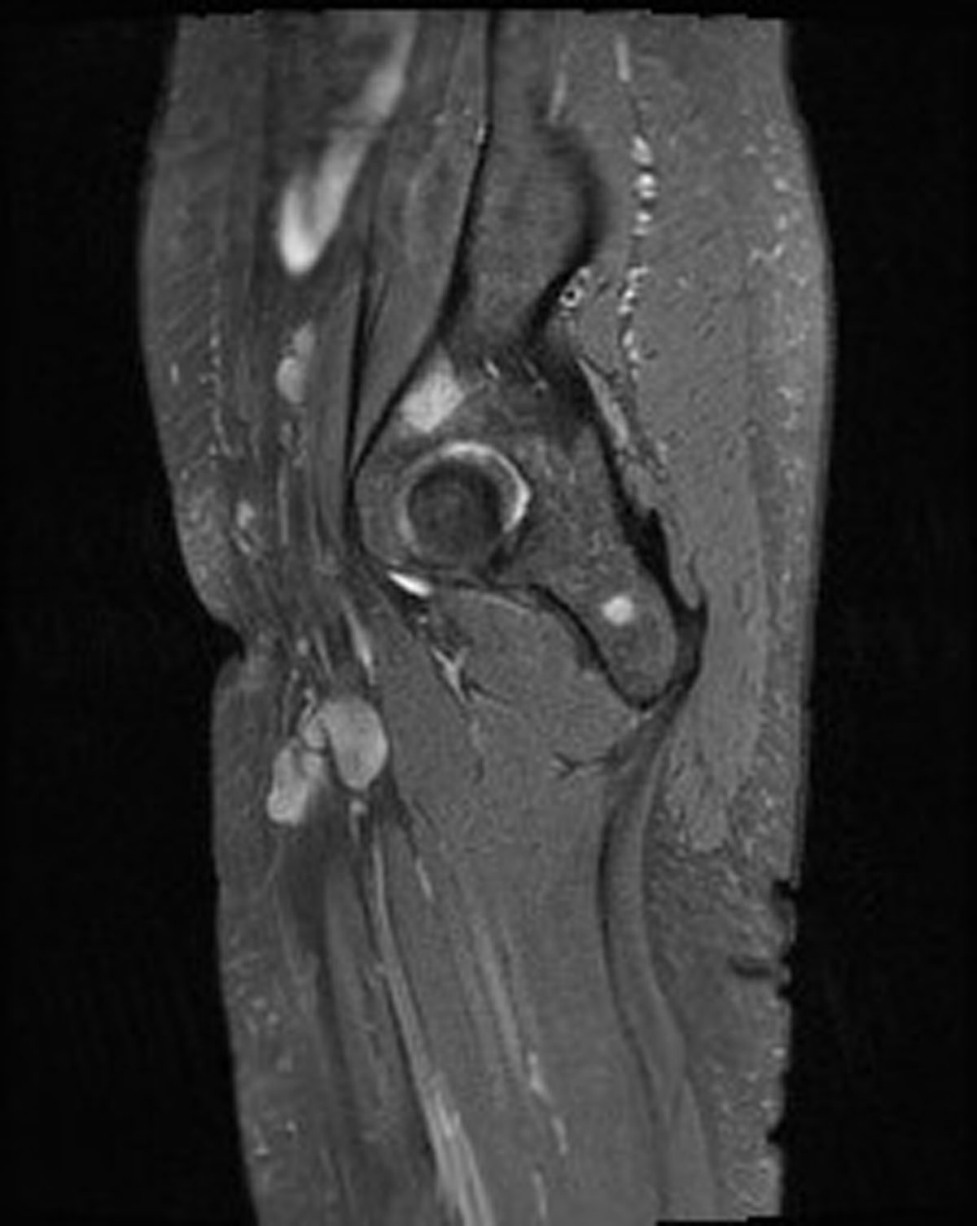
Lesion over Rt acetabulum

## Treatment

Intavenous zolendronic acid helped with bone pain and hypercalcemia. Commenced on azathioprine however unable to tolerate because of headaches. Continued to have active disease and started on infliximab and methotrexate. She improved on this combination treatment and managed to reduce steroids gradually. She is on this treatment for a year and the condition is well controlled.

## Discussion

Granulomatous bone involvement has an overall incidence of 1-13%. The small bones of hands and feet are the most common localizations, while skull, knee, rib, pelvic and sternal localizations are rarely reported [2]. The diagnosis of the sarcoid is based on the clinical presentation, radiological manifestations and histopathological assay. Even though radiographic findings are characteristic, could be challenging to distinguish from bony metastasis. On literature review there are reported case of vertebral sarcoidosis treated with anti TNF agents, our case report suggests infliximab can be effective in treatment of refractory sarcoidosis involving pelvic bone [3,4].

## Conclusion

This is first case reported on successful use of anti-TNF in treatment of multisystem sarcoidosis involving pelvic bone. There is currently limited evidence for biological agents such as infliximab, a monoclonal anti–TNF α antibody in the treatment of multisystem sarcoidosis [1]. Our case supports the need for randomized controlled clinical trials of anti-TNF therapy in refractory systemic sarcoidosis.
